# Regional Collaborations as a Way Forward for Maternal, Newborn and Child Health: The South Asian Healthcare Professional Workshop

**DOI:** 10.3329/jhpn.v28i5.6149

**Published:** 2010-10

**Authors:** Jennifer H. Requejo, Kadidiatou Toure, Zulfiqar Bhutta, Imtiaz Katz, Shahida Zaidi, Andres de Francisco

**Affiliations:** ^1^ Institute for International Programs, Johns Hopkins Bloomberg School of Public Health, 615 North Wolfe Street, Baltimore, MD 21205, USA; ^2^ Partnership for Maternal, Newborn and Child Health, 20 Avenue Appia, CH-1211 Geneva 27, Switzerland; ^3^ Aga Khan University, Stadium Road, Karachi 74800, Pakistan; ^4^ Midwifery Association of Pakistan, 36-C, Street 14, DHA Phase V, Karachi, Pakistan; ^5^ International Federation of Obstetrics and Gynecology, Suite 3–Waterloo Court, 10 Theed Street, London, UK

**Keywords:** Capacity-building, Child health, Child welfare, Healthcare associations, Maternal health, Maternal welfare, Neonatal health, Regional collaboration, Workshops, Asia, South

## Abstract

This article reviews the importance of regional initiatives in the context of global efforts to achieve the Millennium Development Goal 4 and 5 and describes the action-oriented multi-country healthcare professional association (HCPA) workshops organized by the Partnership for Maternal, Newborn and Child Health. The South Asian HCPA workshop served as a catalyst for strengthening the ability of HCPAs in South Asian countries to organize and coordinate their activities effectively, play a larger role in national planning, and collaborate with other key stakeholders in maternal, newborn and child health.

## INTRODUCTION

The third in a series of regional Healthcare Professional Association (HCPA) Workshops, organized by the Partnership for Maternal, Newborn and Child Health (PMNCH), in collaboration with the International HCPAs, was held on 22-25 November 2008 in Dhaka, Bangladesh. The International HCPAs include: International Pediatric Association, International Federation of Gynecology & Obstetrics, International Council of Nurses, International Federation of Midwives, International Pharmaceutical Federation, and Council of International Neonatal Nurses. The workshop brought together representatives of HCPAs from six Asian countries (5 from South Asia, including Afghanistan, Bangladesh, India, Nepal, and Pakistan; and Myanmar from East Asia). The aim of the workshop was to strengthen the ability of the participating HCPAs to promote, design, and implement plans and policies for achieving the Millennium Development Goal (MDG) 4 and 5 [MDG 4 and 5 call for the reduction of child mortality and improvement of maternal health respectively]. Additional objectives included identification of solutions to common challenges to improving service-delivery for maternal, newborn and child health (MNCH) in the region.

The significance and background factors leading to the development of the multi-country HCPA workshops have been described elsewhere (1). These workshops, held to date in the South Asian, East and West African and Middle Eastern regions, are a response to the call-to-action articulated in several high-level meetings and reports about the need for greater participation of HCPAs in the development of approaches for achieving MDG 4 and 5 (2–3). Given that members of HCPAs are skilled professionals responsible for service-delivery, HCPAs are uniquely positioned to provide input on service needs and on strategies for effective translation of plans and policies into clinical practice.

The Multi-country HCPA Workshops are designed as capacity-building exercises to better enable HCPAs in regions with a high burden of maternal and child mortality to organize and engage in advocacy activities and work together to actively contribute to the development of integrated MNCH programmes and policies. Specifically, the workshops consist of two main activities: (a) Sharing of best practices through plenary sessions and panel discussions and (b) group work resulting in the production of country action plans to guide joint HCPA activities over a 1-2-year period. Full documentation of the workshops is available at the PMNCH website (http://www.who.int/pmnch/activities/countries/healthcareprofessionals/en/index.html).

This paper discusses the importance of carrying out regional HCPA activities in South Asia (although Myanmar is in East Asia, it faces a similar burden of disease and obstacles to improving in MNCH) and major workshop outcomes (4–5). The process of developing the country action plans has also been described.

## THE SOUTH ASIAN WORKSHOP: CAPACITY-BUILDING AND COLLABORATION

Concerned countries of the world have passed the mid-point of the target date for reaching MDG 4 and 5, and collective efforts to achieve these goals are urgently needed. Such efforts are particularly critical in South Asia and sub-Saharan Africa where the concentration of maternal and child deaths is the highest and progress towards reduction of mortality is the slowest (6). Precedents have already been set in the South Asian region for successful South-South collaboration. Intraregional trade, for example, is being promoted through the South Asian Association for Regional Cooperation (SAARC). Although there are several regional HCPAs (e.g. paediatrics, obstetricians, and gynaecologists), there is no mechanism in place to promote joint activities to improve the quality of services and health outcomes across the MNCH continuum. The South Asian Workshop provided an opportunity for participants to work towards greater regional collaboration in health and identify and discuss solutions to shared problems in reducing MNC mortality (Box).

Box. **Common bottlenecks to improving MNCH in South Asian countries**

Contextual factors

- Widespread undernutrition and micronutrient deficiencies
- Gender inequities
- High fertility, highest unmet need for family planning, highest rate of births by adolescents
- Rapid growth of urban slums
- Low political prioritization of MNCH
- Conflict and population displacement
- Increasing need for MNCH provision in emergency situations

Health systems factors: supply and demand issues

- Poor and inequitable coverage of proven MNCH interventions
- Human resource crisis (uneven distribution of workforce and insufficient numbers for rural care)
- Weak healthcare referral systems
- Chronic underfunding of the health sector
- Inefficient procurement and supply chain management systems
- Vertical programming
- Low demand for services at the community level

MNCH=Maternal, newborn and child health

The South Asian Multi-country Workshop was designed to strengthen ties across national, regional and international HCPAs; among HCPAs in participating countries; and between HCPAs and other key stakeholders in MNCH, including the Ministry of Health and development partners. The workshop also aimed at developing strategies for HCPAs in the participating countries to align their MNCH-related activities, take advantage of each other's strengths and experiences, and pool resources for the implementation of joint programmes of work (7–10).

Healthcare professionals, such as paediatricians, obstetricians, nurses, midwives, and pharmacists from HCPAs ranging in size, experience, and organizational strength, attended the workshop. Other participants included ministry of health staff from participating countries and representatives of Australian Agency for International Development (AusAID), United States Agency for International Development (USAID), World Health Organization (WHO), United Nations Population Fund (UNFPA), and United Nations Children's Fund (UNICEF).

The first half of the workshop involved sessions on five growth areas for HCPAs, such as organizational strengthening, planning, improvement of service quality, human resources, and advocacy. These sessions created a forum for sharing innovative approaches adopted by the participating HCPAs to build their own capacity and play a role in efforts to achieve MDG 4 and 5. Specific examples are the following:

- The partnership between the Government and the Obstetric and Gynecological Society of Bangladesh to establish a skilled community birth attendant programme
- The participation of the Afghanistan Pediatric Society in the development of national guidelines and strategies for child health, including the introduction of the Integrated Management of Childhood Illness programme
- A joint project between the International Federation of Obstetrics and Gynecology and the Society of Obstetrics and Gynecology in Pakistan, resulting in improved demand for, and availability of, emergency obstetrical care and fistula-treatment services at the community level
- A number of positive examples from India demonstrating how HCPAs can become well-organized, sufficiently resourced, and influential in MNCH-related activities at the national and regional levels
- The Lady Health Worker Program of Pakistan, which has contributed to substantial reduction in stillbirths and maternal mortality through scaling up of community health workers trained in delivery and postnatal care, was also presented as a successful example of human resource management and task shifting.

The plenary sessions facilitated discussions on challenges and opportunities for the further development of South Asian HCPAs in each of the five key growth areas. The major constraints, in addition to those listed in the Box are budget shortfalls, lack of ownership of activities, low motivation among HCPA members, and the ‘siloing’ of HCPA activities, e.g. few collaborative activities across the associations and non-integration of pharmacists and anaesthesiologists into joint projects. Recommendations for strengthening the HCPAs and addressing these constraints included the following:

- Encouraging the HCPAs to adopt a more democratic structure to increase the involvement of members in agenda-setting given that most HCPA initiatives are currently donor-driven
- Developing strategies to increase the participation of HCPAs in efforts to improve the referral structure and implement MNCH-related policies
- Promoting partnerships between the HCPAs and other civil society organizations to increase the effectiveness of advocacy-related activities for greater political prioritization of MNCH, the development of retention schemes and improved management of existing human resources, and the adoption of needed MNCH policies
- Integrating the concept of quality and cultural competence into curriculums and training
- Increasing the involvement of HCPAs in the community-level work to create a higher demand for quality services.

## COUNTRY ACTION PLANS: A STIMULUS TO JOINT WORK ACROSS THE CONTINUUM OF CARE

Although countries in the South Asian region share many similarities, they have distinctive sociocultural frameworks, political systems, languages, and varying degrees of economic development. This diversity suggests that country-tailored strategies for the improvement of MNCH must be prioritized. During the second half of the South Asian Workshop, the country teams designed action plans with feasible and measurable actions that could improve their ability to contribute to the improvement of MNCH in a 1-2-year time span. This process was informed by the best practices shared in the first half of the workshop.

The process of developing the country action plans involved a series of steps: Each team was requested to: (a) arrive at consensus on 2-3 priority intervention areas for achieving MDG 4 and 5, (b) agree on a maximum of three specific activities per selected intervention area, (c) outline the steps required for accomplishing and funding these activities, and (d) select indicators to monitor progress in implementing the action plans.

**Fig. 1.**
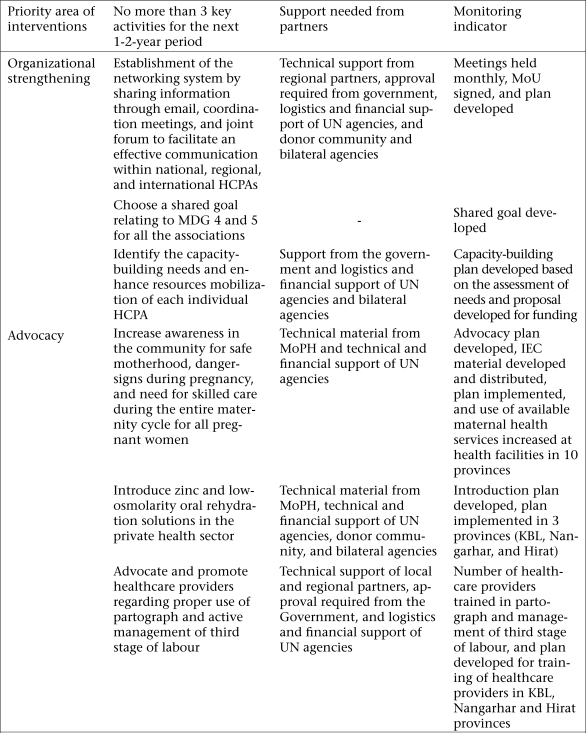
Afghanistan country action plan

The country action plans match the level of development of the national HCPAs and their readiness to undertake collaborative activities. The Afghanistan plan (Fig. 1) emphasizes organizational strengthening because of major capacity-building needs of the country and advocacy activities to raise awareness levels of critical MNCH issues among healthcare providers and community members. In India where many HCPAs are well-established, the plan focuses on incorporating the quality of service into curricula, training, and standards of care and promoting increased collaboration across HCPAs and between HCPAs and the Government. The country action plans for all the participating countries are available on the PMNCH website.

HCPAs=Healthcare professional associations; IEC=Information, education, and communication; KBL=Kabul province; MDG=Millennium Development Goal; MoPH=Ministry of Public Health; MoU=Memorandum of understanding; UN=United Nations

**Fig. 2.**
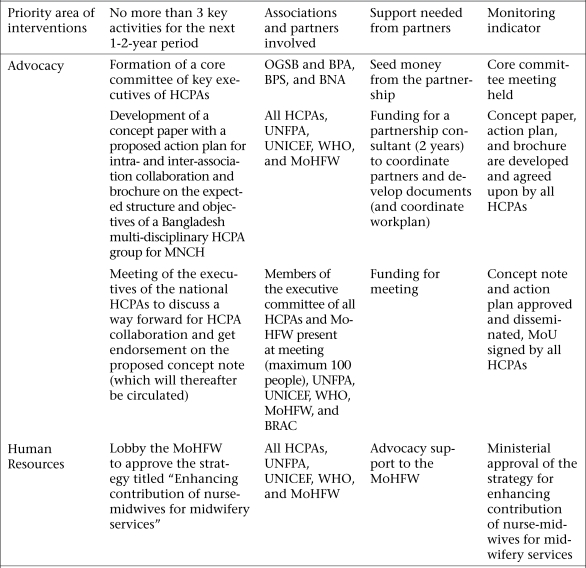

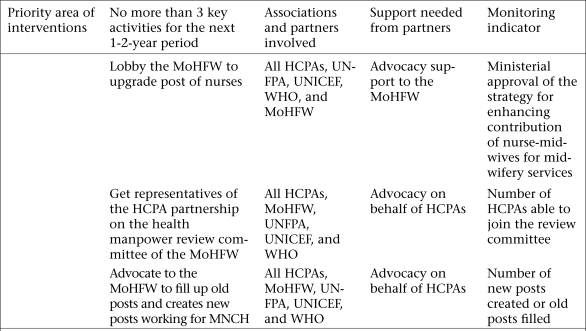
Bangladesh country action plan

The country action plan for Bangladesh is shown in Figure 2. The team agreed to establish a national HCPA partnership for MNCH with representation from 12 key HCPAs and identified advocacy and human resources as the two major gap areas for cross-HCPA work. In the area of advocacy, the plan lists activities to increase coordination across the HCPAs, encourage HCPAs to support the continuum of care, raise greater political commitment to MNCH, and improve healthcare-seeking behaviours at the community level. To address the crisis of human resources, the plan includes activities to push the Government to fill all vacant posts, create needed posts, and increase training opportunities; improve monitoring and supervision of community health workers; and address bottlenecks in the supply chain management system to reduce the frequency of stock-outs.

BNA=Bangladesh Nurses Association; BPA=Bangladesh Paediatric Association; BPS=Bangladesh Perinatal Society; HCPAs=Healthcare professional associations; MNCH=Maternal, newborn and child health; MoHFW=Ministry of Health and Family Welfare; MoU=Memorandum of understanding; OGSB=Obstetrical and Gynaecological Society of Bangladesh; UNFPA=United Nations Population Fund; UNICEF=United Nations Children's Fund; WHO=World Health Organization

The progress reports submitted semi-annually by the teams to the PMNCH showed that the progress in the implementation of the action plans varied across the participating countries. In Afghanistan, the action plan is serving as the collaborative platform for a formal HCPA network established following the workshop. The workshop stimulated other activities in Afghanistan, such as a collaboration between the paediatric and the pharmacy association with the Government to introduce zinc and low-osmolarity oral rehydration solution to the private sector, successful efforts of the obstetrics and gynaecology association to increase training opportunities on emergency obstetric care, and advocacy work by the midwifery association to promote the use of the partograph and active management of the third stage of labour.

The participating associations in India are working to officialize the HCPA network in the country. Most associations in the network have approved a Memorandum of Understanding re-affirming their commitment to MDG 4 and 5 and are using the action plan as the organizing framework for the network's scope of work for the next two years. Other post-workshop collaborative activities include the launching of an advocacy campaign by the Society of Midwives and the White Ribbon Alliance to promote greater political commitment to safe motherhood, the leadership role of the pharmacy association in establishing a working group in the International Pharmaceutical Federation to write a reference paper on the role of pharmacists in MNCH and a presentation delivered by the Federation of Obstetrics and Gynecologists at the annual convention of the Indian Pharmaceutical Association on the importance of pharmacists in achieving the health-related MDGs.

The HCPAs of Pakistan have developed a common vision and action plan. An in-country meeting funded by the UNICEF involving the participation of government representatives and HCPAs resulted in the signing of a Memorandum of Understanding which endorsed the action plan. The action plan has also been presented to the Speaker of the National Assembly, the Ministry of Health, and at national HCPA conferences. The Pakistan team secured support through the Aga Khan Foundation to implement the action plan in selected districts and is seeking funding to bring implementation to scale.

Funding shortfalls, lack of resources, and little communication within the team have proved to be major barriers to the ability of Bangladesh to implement its action plan.

Following the Workshop, the paediatric and obstetric and gynaecology societies in Myanmar initiated the “Strengthening quality reproductive health services of general practitioners” programme to provide training on topics, such as family planning, antenatal and postnatal care, essential newborn care, emergency obstetric care, HIV, prevention of maternal-to-child transmission of HIV, and treatment of sexually transmitted infections. In accordance with the action plan, activities to train another 480 general practitioners are underway, and the medical association is providing training to general practitioners on emergency management of maternal and child health.

## CONCLUSION

The South Asian Multi-country Workshop is part of a broader initiative supported by the PMNCH to increase the capacity of HCPAs to participate in the national-level planning for MNCH and play a more pivotal role in helping countries accelerate progress towards MDG 4 and 5. The South Asian Workshop showed that regional activities can provide a useful platform for fostering stronger ties among the international, regional and national-level HCPAs; the sharing of the best practices; and creating consensus across HCPAs to work together to promote the MNCH continuum of care. Importantly, the process of developing action plans also enabled the country teams to establish an agendum for how they can begin undertaking joint work to achieve their common goal of improving lives of women and children. The format and aims of the workshop are in keeping with growing consensus on the importance of partnerships for the achievement of MDG 4 and 5.

A Fourth Multi-country Workshop was held in Amman, Jordan, in late December 2009. This workshop involved participation from the HCPAs in the Middle Eastern region and followed a similar format. Based on lessons learnt during the South Asian Workshop, greater emphasis is being placed on follow-up activities, such as helping the participating HCPAs find technical and financial support to carry out their action plans. A set of simple questionnaires was introduced in Amman to more systematically assess the immediate and longer-term impact of the workshop on enhancing the capacity of the participating HCPAs to implement their action plans. These tools will measure changes in funding patterns for the HCPAs, levels of coordination within and across the HCPAs, and the relationships between HCPAs and other constituencies, including the Ministry of Health, development partners, and other civil society organizations. These tools will also be used for identifying the barriers and facilitating factors to the ability of HCPAs to become more involved in national-level MNCH planning.

A formal evaluation to assess the impact of the South Asian and African workshops on the activities of HCPAs in the 17 participating countries is scheduled for late 2010. An external evaluation team will carry out the assessment.

## ACKNOWLEDGEMENTS

The authors thank the country team members of Afghanistan, Pakistan, Myanmar, Nepal, Bangladesh, and India for participating in the workshop and providing ongoing information on progress in implementing the country action plans.

The work undertaken to develop this article should be attributed to the Partnership for Maternal, Newborn and Child Health. There was no funding source for the development of the paper.
